# Health Benefits of Green Banana Consumption: A Systematic Review

**DOI:** 10.3390/nu11061222

**Published:** 2019-05-29

**Authors:** Ana Luisa Falcomer, Roberta Figueiredo Resende Riquette, Bernardo Romão de Lima, Verônica C. Ginani, Renata Puppin Zandonadi

**Affiliations:** 1 Faculty of Health Sciences, Department of Nutrition, University of Brasília, Brasilia 70910-900, Distrito Federal, Brazil; anafalcomer@gmail.com (A.L.F.); bernardolima156@gmail.com (B.R.d.L.); vcginani@gmail.com (V.C.G.); 2 Campus Oeste Liliane Barbosa, Department of Nutrition, Instituto de Ensino Superior de Brasília (IESB), Brasilia 72225-315 Distrito Federal, Brazil; rriquette@hotmail.com

**Keywords:** Green banana biomass, green banana flour, unripe banana, health effects

## Abstract

Despite the growing demand for green banana (GB) products, there is no review study regarding their potential health benefits. We aimed to compare the health benefits among different GB products by a systematic review. We researched six electronic databases (PubMed, EMBASE, Scopus, Science Direct, Web of Science, and Google Scholar) from inception to March 2019. We found 1009 articles in these databases. After duplicate removal, we screened 732 articles’ titles and abstracts, and selected 18 potentially relevant studies for full-text reading. We added five records from the reference list of the fully-read articles and seven suggested by the expert. Twelve articles were excluded. In the end, 18 studies were considered for this systematic review. Ten studies were conducted with green banana flour and eight with the green banana pulp/biomass. Most of the GB health benefits studied were related to the gastrointestinal symptoms/diseases, followed by the glycemic/insulin metabolism, weight control, and renal and liver complications associated to diabetes. Only one study did not confirm the health benefit proposed. It is necessary to standardize the GB dose/effect to different age groups and different health effects considering the GB variety and ripeness level. Further studies are necessary to present better detailing of GB product and their health effects considering all the raw-material characteristics.

## 1. Introduction

Fruits are essential components of a healthy diet due to their content of vitamins and minerals, fiber, and beneficial non-nutrient substances as bioactive compounds. The World Health Organization (WHO) recommend ingestion of at least 400 g (about five portions) of fruits and vegetables per day [1]. Low fruit consumption is one of the main risk factors for increased mortality also increases the risk of chronic diseases and poor health quality. Therefore, the regular consumption of fruits can reduce the incidence of some diseases such as diabetes, cardiovascular and gastrointestinal diseases, and some types of cancer [2,3]. 

Banana (Musa sp.) is one of the most cultivated tropical fruit in the world. Worldwide more than 1000 varieties of bananas are produced. The most commercialized is the Musa Cavendish (about 45% of global banana market), due to its high production per hectare and its less prone to damage from environmental changes. The other large variety group of banana is the plantain (that has upwards of 100 cultivars) [4]. The banana varieties production corresponds to about 15% of the world’s total fresh fruit produced [5] reaching about 110 million tons of bananas per year [4]. However, almost one-third of all bananas gathered is lost since the population mostly consume ripe bananas, and it is a climacteric fruit. Ripe bananas are prone to mechanical damage and are perishable during the maturation process, which makes their storage and transport difficult [6]. Almost 20% of banana production is not commercialized due to size and appearance flaws, increasing their loss [7]. Therefore, fruit processing emerged aiming to solve problems such as the weak infrastructure, inadequate transportation, and perishable nature of the production, therefore the grower sustains substantial losses. During the post-harvest glut, the loss is considerable, and often, some of the production must be allowed to rot [8]. The optimization of banana processing has been studied to reduce the production of the waste (annual rejection is about 1/4 of the banana fruit) and to improve the bioavailability and utilization of nutrients available in this fruit, highlighting the use of green banana (GB) products [6,9,10]. 

The consumption of GB products is booming due to their nutritional potential and physiological benefits to human health [10]. Commercial standard color charts classify the stages of banana maturation (Stage 1 = all green, 2 = green with a trace of yellow, 3 = more green than yellow, 4= more yellow than green, 5 = yellow with a trace of green, 6 = full yellow, 7 = full yellow with brown spots) [11,12]. The green banana is commonly used as stages 1 and 2 of maturation (until 9 weeks; L* ranging from 56 to 68, a* ranging from −20 to −15, and b* ranging from 33 to 38) [13,14,15,16,17]. Green bananas seem to be a good source of fibers, vitamins (Vit C, B6, provitamin A), minerals (potassium, phosphorus, magnesium, zinc), bioactive compounds such as phenolic compounds, and resistant starch (RS) [18,19,20,21,22,23,24], potentially contributing to health benefits [10,25,26,27,28,29], classifying GB as functional food [9].

However, people do not usually consume the fresh green banana, mainly due to the typical hardness and its high astringency, caused by the presence of soluble phenolic compounds as tannins [30]. Therefore, studies have been using different GB derivate products such as flour [31,32], and green banana biomass (GBB) [33,34,35]. Despite the growing worldwide demand for GB products [35,36,37,38], there is no review study regarding the potential health benefits of GB and its derivatives. Therefore, this study aimed to compare the health benefits among different GB derivate products by a systematic review.

## 2. Materials and Methods 

Preferred Reporting Items for Systematic Reviews and Meta-Analyses (PRISMA) Checklist [39], and Guidance of the European Food Safety Authority [40] were used to conduct this systematic review.

### 2.1. Protocol and Registration

The protocol study was not recorded in PROSPERO (an international database of prospectively registered systematic reviews in health and social care, welfare, public health, education, crime, justice, and international development, where there is a health related outcome) since this platform is not destined to reviews with food as the main subject.

### 2.2. Eligibility Criteria

#### 2.2.1. Inclusion Criteria

Experimental studies that evaluated the health benefits of green banana consumption were included without language, time, and study restrictions. Despite the absence of language restriction to the abstract, main text, and keywords, the search strategy was performed in English language databases.

#### 2.2.2. Exclusion Criteria

We applied the following exclusion criteria: (i) reviews, letters, conference summaries, case reports, short communications, and books; (ii) studies of green banana mixed with another ingredient; (iii) studies that did not analyze the health benefits of green banana consumption. 

### 2.3. Information Sources

Detailed individual search strategies were developed for each database (Web of Science, Pubmed, Embase, and Scopus). We used the Google Scholar platform to research the gray literature. The final databases search occurred on March 28th, 2019. The lists of references of manuscripts selected for full-text reading were manually examined by two independent researchers for potentially relevant studies that could have been lost during the search of the databases.

### 2.4. Search Strategy

We selected and adapted the appropriate combinations of truncation, and words for the search in each database (Appendix A). We managed all references using Endnote Web and Rayyan software and removed duplicate hits.

### 2.5. Study Selection

We conducted the selection in two phases. In the first phase, two reviewers (ALF and RFRR) independently reviewed the titles and abstracts of all manuscripts identified from databases. The reviewers discarded the articles that did not meet the eligibility criteria. In the second phase, the reviewers (ALF and RFRR) applied the eligibility criteria to the full texts of the selected articles. In the two phases, in cases of disagreement, the issue was discussed until a consensus was obtained. In cases that there was no consensus, the final decision was made by the third reviewer (BRL). The complete text of the manuscripts were considered to the final selection. The RFRR examiner critically evaluated the list of references of the selected studies. After this phase, two reviewers (ALF and RFRR) extracted data from the manuscripts. The third examiner (BRL) and experts (VCG and RPZ) added additional studies.

### 2.6. Data Collection Process

We collected the following information from the studies: Authors and year of publication, country of the research, the aim of the study, methods, primary results, nutritional characteristics evaluated, and the type and maturation level of the banana. Calibration exercises were conducted before starting the review process to guarantee consistency among reviewers.

Three reviewers (ALF, RFRR, and BRL) synthesized the data collected using a standardized table containing: Reference; country; aim; study outline; type of method applied; analysis method; banana product analyzed; nutritional composition; health benefit analyzed; population analyzed; number of samples analyzed (total); and result regarding presence of health benefit (yes/no).

### 2.7. Risk of Bias

We used Meta-analysis of Statistics Assessment and Review Instrument (MASTARI) protocol (Joanna Briggs Institute 2014) to evaluate the risk of bias in the manuscripts. The bias risk assessment instrument included eight questions:

1. Were the analyzed products characterized in detail?
2. Was the method of health benefit association analysis specified?
3. Was the method used certified/validated by Codex and/or AOAC?
4. Was the result of health benefit determined quantitatively?
5. Were the methods of consumption of green banana or sample homogenization of the study samples described?
6. Was the experimental design appropriate?
7. Was the statistical analysis adequate to the purpose of the study?
8. Did the results answer the main question?

The risk of bias was categorized (Appendix B) as “High” when the study reached up to 49% score “yes”; “Moderate”, when the study reached 50% to 69% score “yes”; and “Low”, when the study reached more than 70% score “yes”.

## 3. Results and Discussion

**Figure 1:**
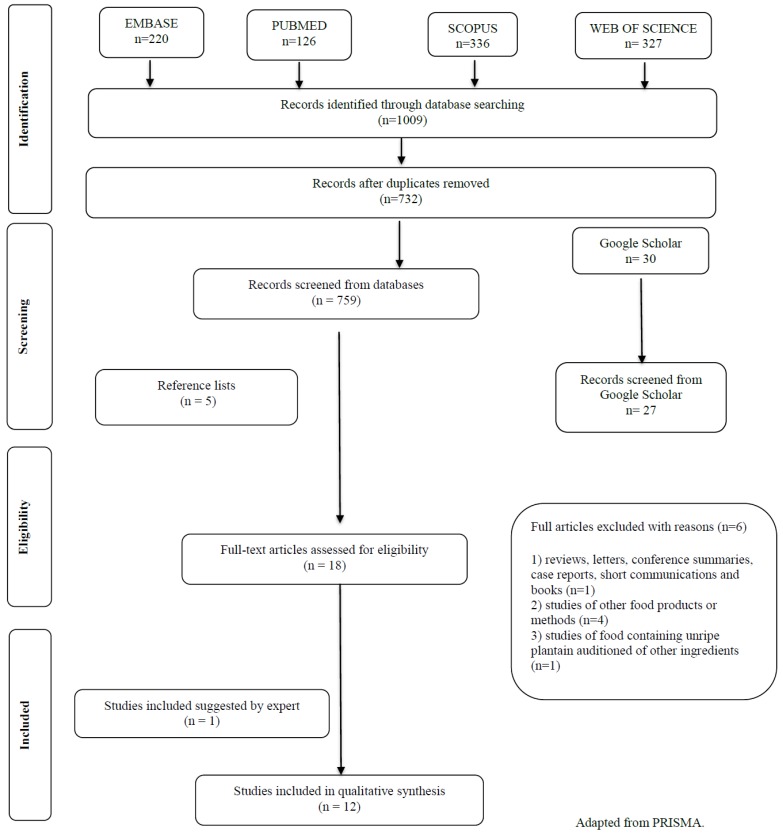
Flow Diagram of Manuscripts screening and inclusion process.

Initially, we identified a total of 1009 articles in the electronic databases. After duplicate removal, we screened the titles and abstracts of 732 articles, and 18 potentially relevant studies were included for full-text reading. An additional five records were selected from the reference list of the fully-read articles and seven suggested by the expert. We excluded 12 articles after fully-reading (Appendix C). Finally, 18 studies met the inclusion criteria and were included in this systematic review. Figure 1 shows the steps of study identification, screening, and inclusion process of the manuscripts.

### 3.1. Studies General Characteristics

The studies occurred in nine different countries: England [41], Sweden [42,43], Bangladesh [44,45,46], Jamaica [47], Venezuela [48], Mexico [49], Brazil [50,51,52,53,54], Nigeria [55], and India [56] (Supplementary file Figure S1) between 1984 and 2019 (Table 1). Almost 40% (*n* = 7) of the studies were conducted in Brazil [50,51,52,53,54], probably due to the Brazilian banana production corresponding for about 15% of the worldwide banana production. Brazil reportedly has one of the highest banana per capita consumption (about 60 kg/year) [4,57]. Latin America countries are the biggest banana exporters, and the European countries and the United States are the biggest importers [58]. It is noteworthy that only 15% of banana production is traded in the international market; the rest is locally consumed, contributing largely to people’s diets [4]. Despite the large consumption of bananas in the United States, none were performed there, probably because the product is imported (with little or no local production) and they have access only to ripe bananas [4]. 

Regarding the ingredients or foods used in the research, ten studies were conducted with green banana flour [41,43,49,50,51,52,53,55,56,59] and eight with the green banana pulp/biomass [42,44,45,46,47,48,54,60]. Most of the studies used green banana flour, probably due to the highest shelf-life and stability of the product compared to the green banana pulp/biomass [61], and this product is well characterized in the scientific literature. However, this product tends to be expensive and not accessible in some countries [10,32,36,62,63,64,65,66,67,68,69,70,71,72], stimulating the utilization of green banana pulp/biomass in some products. Considering the type of research method, 14 of the studies were performed in vivo [41,42,44,45,46,47,48,49,51,52,54,55,59,60], one conducted in vitro [56], and three studies used both in vivo and in vitro methods [43,50,53].

The study population was diverse. Of the in vivo studies, five were performed with rats [41,42,53,55,59] and 12 with humans, five with children and adolescents [44,45,46,48,54] and seven with adults [43,47,49,50,51,52,60], both healthy and unhealthy individuals (persistent diarrhea, constipation, diabetes, overweight, and obesity), showing health benefits in different age groups. Most of the studies analyzed were performed under experimental design, a randomized clinical trial (*n* = 16). The characteristics of the studies in chronological order is detailed in Table 1. 

### 3.2. Nutritional Composition of Green Banana Derivatives, Health Benefits, and Total Amount Used in the Studies

Table 2 presents the type and the amount of green banana used and the composition of green banana products found in each study. In the Alvarez-Acosta et al. [48] study, the authors did not present the composition of the GB product used. Therefore, it is not presented in Table 2.

The studies presented different types of analyses regarding the composition of the green banana product offered (Table 2). It is necessary to emphasize that the food composition like fruits tend to vary according to the soil, climate, banana variety, maturation stage, local of production, and other factors which can explain the significant difference between the results found in the studies regarding the product composition. People tend to use the green banana products due to the claim that they present about of 60–80% of their carbohydrates as indigestible carbohydrates (resistant starch, cellulose, hemicelluloses, and lignin) [30,73]. Also, the green banana flour has been highlighted due to its content of resistant starch, making its use attractive for food preparation where starch is the basis. Resistant starch (RS) has the advantage on food production of having less impact on the sensory properties of the final products (such as better appearance, texture, and mouthfeel), which is favorable for consumer acceptance [74,75]. However, only five studies evaluated RS in which the amount ranged from 5.5 g to 16.6 g of RS in green banana flour [43,50,53]; 34 g to 67 g in green banana starch [49,50]; 1.2 g of RS in cooked green banana flour [43]; and 7.8 g in green banana pulp [54]. 

RS can promote health benefits since it is not hydrolyzed in the digestive tract, and it is fermented in the colon, acting similarly to fibers [73]. RS behaves physiologically like fiber reducing glycemia and consequently helping to prevent or treat type 2 diabetes and decreasing the risk of developing chronic diseases [28]. RS also contributes to the prevention of intestinal diseases; to the blood cholesterol levels reduction [25,76]; to increase the synthesis of B-complex vitamins and mineral absorption; and to improve the immune response and the prevention of the development of intestinal cancer [59,74]. The studies that evaluated the amount of RS in banana flour showed the amount of 5.5 g to 16.6 g of RS in 100 g of green banana flour [43,50,53]. In green banana pulp, the amount was lower (7.8 g/100 g) [54] than found by most of the studies [43,50] with green banana flour. The higher amount of RS in GB flour compared with green banana pulp is expected since GB flour presents low moisture content (1.6–7.6%) [77] due to the dehydration process which concentrates solid components without the use of cooking methods. It is also important to highlight that bananas amylopectin structure is different from corn or potato amylopectin, which present more long chains, and banana starch retrogrades faster than corn or potato starch producing less digestible cooked starch [75,78]. These findings suggest the benefits of green banana products consumption on diseases linked to digestion and glucose/insulin metabolism [47,50,53,56,60].

Regarding fiber content, the studies presented different analysis. Some of them [50,53,56] analyzed total dietary fiber (DF), insoluble fiber (IF), and soluble fiber (SF) in green banana flours (ranging from 5.7 g to 10.3 g of DF; 3.3 g to 53.3 g of IF; and 0.0 g to 12.45 g of SF, respectively). The only study which evaluated dietary fiber in green banana pulp [54] mentioned the amount of 4.4 g /100 g. The other studies presented the amount of crude fiber [47,52,55,56,59] in flour (6.77 g to 65.76 g /100 g) [52,55,56] and in the green banana pulp (0.48 g to 5.52 g /100 g) [47,59]. The fiber content of the green banana pulp also tends to be lower than in the green banana flour (GBF) due to the dehydration of the flour [67,71,77]. Fiber is considered an important ingredient in the formulation of the functional food, due to its beneficial health effects in gut regulation, satiety, appetite control, glycemic regulation, and cancer prevention) [79]. Besides the health effects, they are functional ingredients used to improve the food products physical and structural properties of oil retention, hydration, viscosity, sensory characteristics, and shelf-life [80]. According to Food and Agriculture Organization of United Nations (FAO) [81], the food is considered a source of DF if the DF content is at least 1.5 g of fiber per 100 kcal of food, and high in fiber if it contains 3 g of fiber per 100 kcal of food. Therefore, in some studies, the green banana product could be considered a source of fiber [50,53,54], in addition to the RS content. 

The lipid content in green banana products ranged from 0.43% to 17.59% [49,52,53,55] in green banana flour and it ranged from 0.1% to 0.5% in green banana pulp [42,47,59]. Regarding the protein content, the green banana flour presented from 1.88% to 19.1% [49,52,53,55], and the green banana pulp presented from 1.67% to 2.73% of protein [47,59]. As we mentioned before the large difference between the lipid and protein content in green banana flour is mainly related to the moisture content (that was not mentioned in the studies), and also related to the variety of banana, climate and soil of harvest, steps of flour production, and stage of maturation of the banana. However, only four studies mentioned the stage of maturation of green banana. Three of them used stage 1 (totally green) of maturation [42,50,60], and one referred to the maturation stage as “15 weeks” [49]. The others only mentioned that they used green banana. 

In the production of the green banana product, traditionally stage 1 of banana maturation is used since it presents high antioxidant compounds, high starch, and low sugar contents. These characteristics are essential to promote health benefits that could lead GB as functional food characterization [38,70]. Only the study performed by Eleazu et al. [55] presented the amount of phenolic compounds (7.5 mg GAE/g), since most of the benefits attributed to green banana products consumption are associated with its RS content [28], justifying the few numbers of the studies evaluating the phenolic compounds in the potential health benefits. Phenolic compounds present in fruits are highlighted due to their potential antioxidant effects, which are related to the reduction of the risk of diseases caused by oxidative stress, especially in chronic diseases [82,83,84,85]. Antioxidants can be beneficial in several diseases prevention and treatment since they can play a protective role in the prevention of reactive oxygen species (ROS), mediated damage to the cells and tissues, preventing the harmful action of free-radicals on DNA, proteins, and lipids [86]. Also, they present protective properties against degenerative diseases such as Alzheimer, Parkinson, cancer, and cardiovascular diseases [87]. Eleazu et al. [55] mentioned that phenolics present potential antioxidant activity and that they can act on carbohydrate metabolism involving the inhibition of α-glucosidase and α-amylase (enzymes in charge of digestion of dietary carbohydrates to glucose). The authors also mentioned that GBs higher phenolic amount incorporated to the feed compared with the standard rat feed could explain the higher antidiabetic action found in the study [55]. 

Anyasi et al. [9] studied the phenolic profile of GBF. Of the phenolic compounds examined by the authors, catechin, gallic acid, and epigallocatechin were not detected, different from the data found in other studies [88,89] that indicated the presence of gallic acid and catechin in GB obtained from other countries. The flavonoids epicatechin and myricetin 3-O-rhamnosyl-glucoside were detected in different concentrations in GB [9]. It is necessary to mention that, according to Gallani [90], the total phenolic content in ripened banana decreased when stored in refrigerated conditions (4 ºC) for 15 days. The author stated that the total phenolic content could be influenced by many factors such as genotype, harvest time, and growing location. Another study [21] showed that the production of GBB, when cooked for 5 min, presented higher levels of phenolic compounds (*p* < 0.05) compared with the GBB cooked for 10 min. Refrigerated storage maintained the content of phenolics (*p* < 0.05). Since the authors used high temperatures to cook the GB samples, the activity of the enzyme related to the degradation of the polyphenolic compounds in phenolic compounds in bananas were inactivated, reducing the chance that total phenolic contents decrease during cold storage. Since we found only one study that evaluated GB phenolic compounds and their potential health benefit [55], further studies should be conducted to elucidate the mechanism and confirm this potential correlation.

Among the health benefits studied using green banana products, most of them were related to the gastrointestinal symptoms/diseases [41,42,43,44,45,46,48,54], followed by the glycemic/insulin metabolism [47,50,53,56,60], weight control [49,51,52,56], and renal and liver complications associated to diabetes [55,59]. Only one study did not confirm the health benefit proposed by the study [52]. It is important to emphasize that all the studies mentioned RS and fiber content of green banana as the main components to promote health benefits, despite the mention of phenolics and other potentially bioactive compounds.

All the studies performed with children used green banana pulp/biomass [44,45,46,48,54]. The dose used ranged from 30 g (in total) [54] to 30 g per kg of body weight [44,45]. One study did not use this amount of green banana pulp in grams [46]. They referred the amount of GB as: For children aged 6–12 months, the recommended quantity ranged from one-half to one fruit daily; for 12–24 months, one to two fruits daily; and for 24–36 months, three fruits per day [46]. For adults, the studies performed with GBF used different amounts (8 g of GBF [60]; 20–36.3 g [51,52]; 24 g [49]; 30 g [43]; 54–81 g [50]). Only one study [47] conducted with adults used green banana pulp (GBP). The amount used in the study ranged from 225 g to 260g of GBP [47]. In the studies conducted with rats, the daily dose ranged from 14 g [41] to 17 g [53] of green banana flour. When used the green banana pulp in rats, the dose was 1.3 g [42].

It is important to emphasize the need to standardize some relevant points as the dose/effect to different age groups and different health effects considering the variety of the green banana, the ripeness level. It is also relevant to study the health effects of green banana products when used as an ingredient of a potential “functional food” after the production of the meal, in which can be included manipulation and cook steps. 

In addition to the aspects discussed regarding GB and health, there is a consensus about the relationship between diet, environmental sustainability, and human health. Agriculture presents increasingly global impacts on health, sustainability, diet, and environment [91]. As we mentioned before, the possibility of reduction of banana production waste/lost can significantly impact on health, and it was mentioned by most of the studies evaluated. In addition to the GB, other nonconventional food discarded as wastes from the banana production can be explored, such as banana pseudostem and banana flower, aiming to reduce the environmental damage and improving the sustainability [86] and contributing to the nutritional quality of the diet and reducing the hunger to the low-income population.

### 3.3. Risk of bias

We found heterogenicity between the studies: Eighteen had a low risk of bias and two had moderate risk. Three studies did not present the characterization of the products analyzed in detail [41,42,54]. Moreover, all studies specified the methods of analysis which were approved by the Codex Alimentariusand/or AOAC. All the studies answered its main question with the quantitative and well-described presentation of the health benefit analyzed (Table 3).

## 4. Conclusions

Overall, the studies showed the health benefits using green banana products, most of them related to the gastrointestinal symptoms/diseases, followed by the glycemic/insulin metabolism, weight control, and renal and liver complications associated to diabetes, most of the studies using green banana flour. The children group studies showed that green banana pulp influenced both diarrhea and constipation improvement. In healthy adults’ group, GBF increased satiety and influenced glucose homeostasis, as same as the GBP and GB starch. Considering type 2 diabetes adults, studies showed a reduction of body weight and increase insulin sensitivity with GBF consumption. Among overweight women, GBF consumption improved anthropometric (weight and body composition), lipid profile, and inflammatory parameters. However, there is no standardization regarding dose/effect to different age groups and different health effects considering the variety of the green banana, and the ripeness level. It is also important to emphasize that few studies well characterize the chemical composition of the green banana product used. Further studies are relevant to evaluate if the health effects of green banana products remain when used as an ingredient of a potential “functional food” after the production of the food product.

Therefore, it is essential to present better detailing of the GB product associated with their health effects considering all of the raw-material characteristics, including production, storage, dose-response, and chemical characteristics. Considering the relationship between diet, environmental, economy, sustainability, and human health, the possibility of reduction of banana production waste/lost can probably present a significant impact on health. In addition to the GB, other nonconventional food from the banana production can be explored, aiming to reduce the environmental damage and improving the sustainability, contributing to the nutritional quality of the diet and reducing the hunger to a low-income population.

## Figures and Tables

**Table 1:** Descriptive characteristics and outcomes of interest of the included studies.

Author/year	Country	Aim	Study Outline	Analysis Method	GB* Product (GBF**, GBP*** or GBB ****) AND Health Benefit Analyzed	Population Analyzed	Heath Benefit Confirmation (Yes or No)
Best, Lewis and Nasser (1984) [41]	England	To check the anti-ulcerogenic activity of the unripe plantain banana.	Experimental study, randomized clinical trial	In vivo Extracts of Banana flour were assessed for biological activity against ulceration (prophylactic or curative).	GBF Exercise anti-ulcerogenic activity	14 male rats with ulcer	Yes
Dunjic et al. (1993) [42]	Sweden	To evaluate the protective capacities of GB and their potentially active against acute and chronic gastric mucosal lesions.	Experimental study, randomized clinical trial	In vivo GBP was mixed with saline or pectin and phosphatidylcholine solution and given by gavage, as a pretreatment in a single dose.	GBP Protection of gastric mucosa against experimentally induced injuries in rats	121 male rats with an injury to the stomach	Yes
Rabbani et al. (2001) [44]	Bangladesh	To evaluate the therapeutic effects of GB in children with persistent diarrhea.	Experimental study, randomized clinical trial	In vivo In a double-blind trial, boys were randomly given a rice-based diet containing either 250 g/L of cooked GB or 4 g/kg pectin or the rice-diet alone (control), for seven days. Stool weight and consistency, the frequency of vomiting and purging, and duration of illness were measured.	GBP Control of persistent diarrhea	62 boys (age 5–12 months) with persistent diarrhea	Yes
Langkilde, Champ, and Andersson (2002) [43]	Sweden	To evaluate how RS***** influences small-intestinal absorption of nutrients, sterol metabolism and colonic fermentation	Experimental study, randomized clinical trial	In vitro e in vivo 10 ileostomy subjects received a controlled diet with 30 g raw GBF or cooked GBF in random order. After, 7 ileostomy subjects received a plant-polysaccharide-free diet with the addition of 30 g GBF.	GBF Knowledge of how RS influences small-intestinal absorption of nutrients, sterol metabolism and colonic fermentation	10 ileostomized adults (male 50%; age 28–70 years)	Yes
Rabbani et al. (2004) [45]	Bangladesh	To evaluate whether the effects of GB and pectin were associated with improvement of the small bowel mucosal permeability in children with persistent diarrhea	Experimental study, randomized clinical trial	In vivo Boys with persistent diarrhea (14 days) received week’s treatment with a rice-based diet containing either cooked GB, pectin, or rice diet alone. Intestinal permeability was assessed before and after treatment.	GBP Control of persistent diarrhea	57 boys (5–12 months) with persistent diarrhea (14 days)	Yes
Bahado-Singh et al. (2006) [47]	Jamaica	To determine the Glicemic Index (GI) of some commonly eaten Caribbean carbohydrate-rich foods, processed by different traditional cooking methods.	Experimental study, randomized clinical trial	In vivo GI values for 14 commonly eaten carbohydrate-rich foods processed by various methods were determined using ten healthy subjects. Pure glucose was used as the standard with a GI value of 100.	GB Knowledge of GB glycemic Index. GB presented Low glycemic index when boiled or fried.	10 healthy adults (non-diabetic, 18–40 years)	Yes
Álvarez-Acosta et al. (2009) [48]	Venezuela	To evaluate the beneficial effects of GB based diet on stool volume, frequency and weight gain as compared with a traditional yogurt-based diet in children with persistent diarrhea.	Experimental study, randomized clinical trial	In vivo Prospective, in-hospital controlled trial in children who had experienced 14 days of persistent diarrhea. Two groups of 40 individuals received isocaloric (100 kcal/kg/d) diets: 1) a-week treatment consisting of a 50 g/L of cooked GB-based diet. 2) The control group was fed on a yogurt-based diet.	GBP Control of persistent diarrhea	80 children of both gender with ages ranging from 1 to 28 months, with persistent diarrhea (14 days).	Yes
Rabbani et al. (2010) [46]	Bangladesh	To determine the effectiveness of adding GB to conventional household diets in the management of childhood diarrhea (reducing the duration)	Experimental study, randomized clinical trial	In vivo Individuals were randomly assigned to either a standard care group or standard care plus GB group (mothers were instructed to add a cooked GB to the diets of diarrheal children. Through a village-based surveillance system, diarrheal morbidity data (severity, duration, and compliance) were collected for 14 days.	GBP Treatment of diarrhea acute and prolonged in children	2968 Bangladeshi rural children 6–36 months old with diarrhea	Yes
Ble-Castillo et al. (2010) [49]	Mexico	To compare the effects of native banana starch (NBS) and soy milk (control) on body weight and insulin sensitivity in obese type 2 diabetics	Experimental study, randomized clinical trial	In vivo Subjects undertook two phases of 4-week supplementation with native banana starch (NBS) or soy milk. Were analyzed: glucose, cholesterol, LDL, HDL-cholesterol and triglycerides, Insulin, Glycated hemoglobin (HbA1c) and Insulin Resistance.	GBF Reduction of body weight and increase insulin sensitivity in obese type 2 diabetics.	30 patients with type 2 diabetes, obese, aged between 40 and 60 years	Yes
Menezes et al. (2010) [50]	Brazil	To study the in vitro colonic fermentation profile of unavailable carbohydrates of two different kinds of GBF and to evaluate their postprandial glycemic responses.	Experimental study, randomized clinical trial	In vitro and in vivo The fermentability of the flours was evaluated by different parameters, using rat inoculum, as well as the glycemic response produced after the ingestion by healthy volunteers.	GBF, GBB, and GB starch To present high in vitro fermentability without increasing postprandial glycemic response in healthy subjects	18 healthy adults aged 22–40 years	Yes
Silva et al. (2014) [51]	Brazil	To evaluate the effects of GBF consumption on anthropometric and biochemical parameters in overweight women.	Experimental study, randomized clinical trial	In vivo The GI of GBF in the study was determined. The effects of consumption of 20 g of GBF/day on weight, body mass index, blood pressure, waist and hip circumference, body composition, hemoglobin, lipid profile, glucose, insulin, insulin resistance, liver function, and energy intake were evaluated in overweight women for 45 days.	GBF Improve anthropometric and biochemical parameters with GBF consumption	25 women adults overweight	Yes
Silva et al. (2015) [52]	Brazil	To analyze the effects of consumption of GBF in body weight, lipid profile, inflammatory parameters and food consumption in overweight adult women.	Experimental study, randomized clinical trial	In vivo 25 adult overweight women consumed daily 20g of GBF for 45 days. The study protocol included anthropometric measurements, body composition, food intake, lipid profile, and determination of serum inflammatory parameters.	GBF The consumption of GBF alters weight, body composition, lipid profile, and inflammatory parameters.	25 women adults overweight	No
Eleazu and Okafor (2015) [55]	Nigeria	To investigate the effect of GB on markers of hepatic dysfunction in streptozotocin-induced diabetic rats.	Experimental study, randomized clinical trial	In vivo Blood glucose; relative liver weight; relative kidney weight; relative heart weight; relative pancreatic weight; AST, ALT, and alkaline phosphatase; serum amylase, lipase, total, and conjugated bilirubin; and chemical analysis of the test feed were determined using standard techniques.	GBF Amelioration of renal and liver complications arising from diabetes mellitus	42 diabetic and 6 non-diabetic rats	Yes
Dan et al. (2015) [53]	Brazil	To evaluate the effect of the colonic fermentation of unavailable carbohydrates from unripe banana (mass - UBM - and starch - UBS) over parameters related to glucose and insulin response in rats.	Experimental study, randomized clinical trial	In vivo and in vitro Wistar male rats were fed either a control diet, a UBM diet (5 % resistant starch - RS) or a UBS diet (10 % RS) for 28 days. In vivo (oral glucose tolerance test) and in vitro (cecum fecal fermentation, pancreatic islet insulin secretion) analyses were performed.	GBF, and GB starch Promote colonic fermentation and influence glycemic control, improving insulin sensitivity in rats.	48 healthy male rats	Yes
Sardá et al. (2016) [60]	Brazil	To evaluate the impact of regular, but non-daily, intake of RS from GBF on parameters related to hunger/satiety and glucose homeostasis in healthy volunteers.	Experimental study, randomized clinical trial	In vivo Healthy volunteers consumed GBF, rich in resistant starch (5 g/8 g GBF), nondaily (3 times a week) for six weeks. The parameters hunger and satiety were evaluated by the visual analog scale and area under the curve of ghrelin and peptide YY hormones.	GBF Decrease hunger, increase satiety, and glucose homeostasis	22 healthy adults (27.6 ± 5.1 years)	Yes
Silva et al. (2016) [59]	Brazil	To investigate the effects of a GBP diet on the oxidative damage from type 1 diabetes mellitus (DM)	Experimental study, randomized clinical trial	In vivo Formulations containing 25, 50, and 75% of GBP were included in a 12-week diet of Wistar rats with type 1 DM. They evaluated preventing oxidative damage in kidneys and liver homogenates of rats were evaluated using the TBARS and DNPH assay fasting glycemia, fructosamine levels, renal function, liver function, and lipid profile in the serum of rats.	GBP To prevent oxidative damage in liver and kidney and improves biochemical parameters in type 1 diabetic rats	60 rats with and without diabetes	Yes
Arun et al. (2017) [56]	India	To evaluate the antidiabetic potential of Musa paradisiaca (plantain)	Experimental cross-sectional study	In vitro Were analyzed soluble and insoluble dietary fiber, glucose and cholesterol adsorption capacity and the ethyl acetate and methanol extracts were analyzed for phenolics content, antioxidant activities, antidiabetic, and cardiovascular protection efficacy.	GBF Ameliorates type 2 diabetes and associated cardiovascular risks	-	Yes
Cassettari et al. (2019) [54]	Brazil	To evaluate the effect of combinations of GBB and laxatives in children and adolescents with chronic constipation	Prospective, interventional, randomized clinical study	In vivo It was a randomized study of 80 children and adolescents with functional constipation divided into 5 groups: (1) GBB alone; (2) GBB plus PEG 3350 with electrolytes; (3) GBB plus sodium pyrosulfate; (4) PEG 3350 with electrolytes alone; and (5) sodium picosulfate alone.	GBP To ameliorate the symptoms of chronic intestinal constipation and decrease the use of laxatives.	80 children and adolescents (5-15 years) with functional constipation	Yes

* GB: Green banana product; ** GBF: Green banana flour; *** GBP: Green banana pulp; **** GBB: Green banana Biomass; ***** RS: Resistant starch

**Table 2:** Type and the amount of green banana and the composition of green banana products found in each study.

Author	Type of Green Banana (GB)	Carboydrates (%)	Protein (%)	Lipid (%)	Ash (%)	Crude Fiber (%)	Dietary Fiber (DF) (%)	Insoluble Fiber (%)	Soluble Fiber (%)	Total Starch (%)	RS ^c^ (%)	Daily Dose (g)
**GREEN BANANA FLOUR**												
Best et al. [41]	Musa cavendish and Mondan	-	-	-	-	-	-	-	-	-	-	1) Prophylaxy: 5 g 2) Curative: 7 g
Langkilde et al. [43]	RBF ^e^ and CBF ^f^	-	-	-	-	-	-	-	-	-	RBF: 16.3 CBF: 1.2	30 g
Ble-Castillo et al. [49]	Musa cavendish AAA	60 DB ^d^	1.9 DB	0.4 DB	0.8 DB	-	-	-	-	-	34	24 g
Menezes et al. [50] ^a^	Musa paradisiaca	-	-	-		-	UBM ^g^: 10.3 UBS ^h^: 1.3	UBM: 5.8 UBS: 0.0	UBM: 4.5 UBS: 1.3	UBM: 61.6 UBS: 83.8	UBM: 8.2 UBS: 67.0	59 g de UBS 81 g de UBM
Silva et al. [51]	-	-	-	-	-	-	-	-	-	-	-	20 g/day (45 days)
Silva et al. [52]	-	65	5	0.5	-	11	-	-	-	-	-	20
Eleazu and Okafor [55] ^b^	Musa paradisiaca	55.6	14.7	17.59	-	6.77	-	-	-	-	-	-
Dan et al. [53]	Musa paradisiaca	UBM ^g^: 63.7 UBS ^h^: 64.8	UBM: 19.1 UBS: 17.8	UBM: 6.8 UBS: 7.0	UBM: 2.9 UBS: 4.6	-	UBM: 6.6 UBS: 5.7	UBM: 3.3 UBS: 5.7	UBM: 3.3 UBS: 0.0	UBM: 42 UBS: 42.8	UBM: 5.5 UBS: 10.6	UBS: 5 g of RS/100 g of diet UBM: 10 g of RS/100 g of diet
Sardá et al. [60]	Musa acuminata, Cavendish	-	-	-	-	-	-	-	-	-	-	8 g
Arun et al. [56]	Musa paradisiaca	-	-	-	-	65.76	-	53.31	12.45	-	-	-
GREEN BANANA PULP OR BIOMASS												
Dunjic et al. [42]	Musa Cavendish	-	-	0.1 DB	-	-	-	-	-	-	-	1.3 g
Rabbani et al. [44]	Musa paradisiaca sapientum	-	-	-	-	-	-	-	-	-	-	250 g
Rabbani et al. [45]	Musa paradisiaca sapientum	-	-	-	-	-	-	-	-	-	-	30 g per kg of body weight
Bahado-Singh et al. [47]	Green banana (GB) Green plantain	GB: 25.6 Green plantain: 28.4	GB: 1.42 Green plantain: 1.7	GB: 0.3 Green plantain: 0.2	GB: 0.9 Green plantain: 1.1	GB: 0.6 Green plantain: 0.5	-	-	-	-	-	GB: 225.23 g Green plantain: 259.20 g
Rabbani et al. [46]	-	-	-	-	-	-	-	-	-	-	-	1) Age 6–12 months: one-half to one full fruit per day; 2) Age 12–24 months: 1–2 fruits 3) Age 24–36 months: 3 fruits
Silva et al. [59]	Musa silver variety	-	2.7	0.5	4.5	5.5	-	-	-	-	-	-
Cassettari et al. [54]	Musa spp. AAA	-	-	-	-	-	4.4	-	-	-	7.8	30 g

^a^ Evaluated available starch (%): UBM: 53.2 ± 1.2; UBS: 16.8 ± 0.9; ^b^ Evaluated phenolic compounds (7.50 ± 1.73 mg GAE/g); ^c^ RS: Resistant starch; ^d^ DB: Dry basis; ^e^ RBF: Raw green banana flour; ^f^ CBF: Cooked Banana flour; ^g^ UBM: Unripe banana mass (UBM); ^h^ UBS: Unripe banana starch.

**Table 3:** Summary of risk of bias assessment.

Reference	Risk of Bias	Risk Percentage
Best, Lewis, and Nasser (1984) [41]	Moderate	**66.6%**
Dunjic et al. (1993) [42]	Moderate	**66.6%**
Rabbani et al. (2001) [44]	Low	**100%**
Langkilde, Champ, and Andersson (2002) [43]	Low	**100%**
Rabbani et al. (2004) [45]	Low	**100%**
Bahado-Singh et al. (2006) [47]	Low	**100%**
Alvarzs-Acosta et al. (2009) [48]	Low	**100%**
Rabbani et al. (2010) [46]	Low	**100%**
Ble-Castillo et al. (2010) [49]	Low	**88.8%**
Menezes et al. (2010) [50]	Low	**100%**
Silva et al. (2014) [51]	Low	**100%**
Eleazu and Okafor (2015) [55]	Low	**88.8%**
Dan et al. (2015) [53]	Low	**88.8%**
Silva et al. (2015) [52]	Low	**100%**
Sardá et al. (2016) [60]	Low	**100%**
Silva et al. (2016) [59]	Low	**100%**
Arun et al. (2017) [56]	Low	**100%**
Cassettari et al. (2019) [54]	Low	**88.8%**

## Supplementary Materials

The following are available online at https://www.mdpi.com/2072-6643/11/6/1222/s1, Figure S1: Distribution of studies within countries.

## Appendix A.

**Table A1:** Database search strategy.

Database	Search (September 17, 2018)
PubMed	(“green banana” OR “banana resistant starch” OR “green banana fruit” OR “green banana pulp” OR “unripe banana” OR “unripe plantain” OR “banana biomass” OR “green banana biomass” OR “banana pulp” OR “green banana puree” OR “banana pulp” OR “plantain” OR “unripe plantain” OR “banana puree” OR “plantain pulp” OR “plantain puree” or “green banana flour” OR “banana flour” OR “plantain flour” OR “Musa AAB group” OR “unripe banana resistant starch”)) AND (“health” OR “healthy” OR “welfare” OR “life quality” OR “functional property” OR “functional properties” OR “benefit” OR “benefits” OR “beneficial property” OR “beneficial properties” OR “welfare” OR “health improvement” OR “improvements” OR “well-being” OR “health increase” OR “recovery” OR “health enhance” OR “beneficial effects”)
EMBASE	(‘green banana’ OR ‘banana resistant starch’ OR ‘green banana fruit’ OR ‘green banana pulp’ OR ‘unripe banana’ OR ‘banana biomass’ OR ‘green banana biomass’ OR ‘green banana puree’ OR ‘banana pulp’ OR ‘plantain’ OR ‘unripe plantain’ OR ‘banana puree’ OR ‘plantain pulp’ OR ‘plantain puree’ OR ‘green banana flour’ OR ‘banana flour’ OR ‘plantain flour’ OR ‘musa aab group’ OR ‘unripe banana resistant starch’) AND (‘health’ OR ‘healthy’ OR ‘life quality’ OR ‘functional property’ OR ‘functional properties’ OR ‘benefit’ OR ‘benefits’ OR ‘beneficial property’ OR ‘beneficial properties’ OR ‘welfare’ OR ‘health improvement’ OR ‘improvements’ OR ‘well-being’ OR ‘health increase’ OR ‘recovery’ OR ‘health enhance’ OR ‘beneficial effects’)
Scopus	( ( ALL ( ‘ green AND banana’ OR ‘ banana AND resistant AND starch’ OR ‘ green AND banana AND fruit’ OR ‘ green AND banana AND pulp’ OR ‘ unripe AND banana’ OR ‘ banana AND biomass’ OR ‘ green AND banana AND biomass’ ) ) OR ( ALL ( ‘ green AND banana AND puree’ OR ‘ banana AND pulp’ OR ‘ plantain’ OR ‘ unripe AND plantain’ OR ‘ banana AND puree’ OR ‘ plantain AND pulp’ OR ‘ plantain AND puree’ OR ‘ green AND banana AND flour’ ) ) OR ( ALL ( ‘ banana AND flour’ OR ‘ plantain AND flour’ OR ‘ musa AND aab AND group’ OR ‘ unripe AND banana AND resistant AND starch’ ) ) ) AND ( ( ALL ( ‘ health’ OR ‘ healthy’ OR ‘ life AND quality’ OR ‘ functional AND property’ OR ‘ functional AND properties’ OR ‘ benefit’ OR ‘ benefits’ OR ‘ beneficial AND property’ OR ‘ beneficial AND properties’ OR ‘ welfare’ ) ) OR ( ALL ( ‘ health AND improvement’ OR ‘ improvements’ OR ‘ well-being’ OR ‘ health AND increase’ OR ‘ recovery’ OR ‘ health AND enhance’ OR ‘ beneficial AND effects’ ) ) )
Web of Science	(((“green banana” OR “banana resistant starch” OR “green banana fruit” OR “green banana pulp” OR “unripe banana” OR “unripe plantain” OR “banana biomass” OR “green banana biomass” OR “banana pulp” OR “green banana puree” OR “banana pulp” OR “plantain” OR “unripe plantain” OR “banana puree” OR “plantain pulp” OR “plantain puree” or “green banana flour” OR “banana flour” OR “plantain flour” OR “Musa AAB group” OR “unripe banana resistant starch”)) AND (“health” OR “healthy” OR “welfare” OR “life quality” OR “functional property” OR “functional properties” OR “benefit” OR “benefits” OR “beneficial property” OR “beneficial properties” OR “welfare” OR “health improvement” OR “improvements” OR “well-being” OR “health increase” OR “recovery” OR “health enhance” OR “beneficial effects”))
Google Scholar	(green banana and health benefits)

## Appendix B.

**Table A2:** Quality criteria of the studies selected for the systematic review about health benefits associated with ingestion of green banana.

Reference	Were the Analyzed Products and Meals Characterized in Detail?	Was the Method of Health Benefit Association Analysis Specified?	Was the Method Used Certified/Validated by Codex and/or AOAC?	Was the Result of Health Benefit Determined Quantitatively?	Were the Methods of Consumption of Green Banana or Sample Homogenization of the Study Samples Described?	Was the Experimental Design Appropriate?	Was the Statistical Adequate to the Purpose of the Study?	Did the Results Answer the Main Question?	Were the Health Benefits or Its Absence Well Described?	Percentage of Positive Answers (Yes) for Each Study that Attained the Quality Criteria
**Best, Lewis and Nasser (1984) [41]**	N	Y	Y	Y	N	Y	N	Y	Y	**66.6%**
**Dunjic et al. (1993) [42]**	N	Y	Y	Y	N	Y	N	Y	Y	**66.6%**
**Rabbani et al. (2001) [44]**	Y	Y	Y	Y	Y	Y	Y	Y	Y	**100%**
**Langkilde, Champ and Andersson (2002) [43]**	Y	Y	Y	Y	Y	Y	Y	Y	Y	**100%**
**Rabbani et al. (2004) [45]**	Y	Y	Y	Y	Y	Y	Y	Y	Y	**100%**
**Bahado-Singh et al. (2006) [47]**	Y	Y	Y	Y	Y	Y	Y	Y	Y	**100%**
**Alvarez-Acosta et al. (2009) [48]**	Y	Y	Y	Y	Y	Y	Y	Y	Y	**100%**
**Rabbani et al. (2010) [46]**	Y	Y	Y	Y	Y	Y	Y	Y	Y	**100%**
**Ble-Castillo et al. (2010) [49]**	Y	Y	Y	Y	N	Y	Y	Y	Y	**88.8%**
**Menezes et al. (2010) [50]**	Y	Y	Y	Y	Y	Y	Y	Y	Y	**100%**
**Silva et al. (2014) [51]**	Y	Y	Y	Y	Y	Y	Y	Y	Y	**100%**
**Eleazu and Okafor (2015) [55]**	Y	Y	Y	Y	Y	N	Y	Y	Y	**88.8%**
**Dan et al. (2015) [53]**	Y	Y	Y	Y	N	Y	Y	Y	Y	**88.8%**
**Silva et al. (2015) [52]**	Y	Y	Y	Y	Y	Y	Y	Y	Y	**100%**
**Sardá et al. (2016) [60]**	Y	Y	Y	Y	Y	Y	Y	Y	Y	**100%**
**Silva et al. (2016) [59]**	Y	Y	Y	Y	Y	Y	Y	Y	Y	**100%**
**Arun et al. (2017) [56]**	Y	Y	Y	Y	Y	Y	Y	Y	Y	**100%**
**Cassettari et al. (2019) [54]**	N	Y	Y	Y	Y	Y	Y	Y	Y	**88.8%**

Legend: Y—Yes; N—No; U—Unclear; NA—Not Applicable.

## Appendix C.

**Table A3:** Full-text articles excluded, with reasons.

Author, Year	Reason for Exclusion
Brakohiapa et al. [92]	2
Clark and Manini [93]	2
Rabbani et al. [94]	1
Perera et al. [95]	3
Alegbejo and Ameh [96]	2
Fahrasmane, Parfait and Aurore [97]	1
Cassettari et al. [98]	1
Silva et al. [99]	1
Apostolopoulos et al. [38]	1
Putu, Fiastuti and Kurniarobbi [100]	2
Vieira et al. [101]	3
Izar et al. [102]	1

Legend—Exclusion criteria: 1—reviews, letters, conference summaries, case reports, short communications and books (*n* = 3). 2—full article not found (*n* = 7). 3—article does not focus on analyzing the green banana health benefits (*n* = 2).
