# Supplementary figures and images for: Health Benefits of Green Banana Consumption: A Systematic Review
Source: Nutrients. 2019 May 29;11(6):1222. doi: 10.3390/nu11061222 (PMC6627159; doi:10.3390/nu11061222)

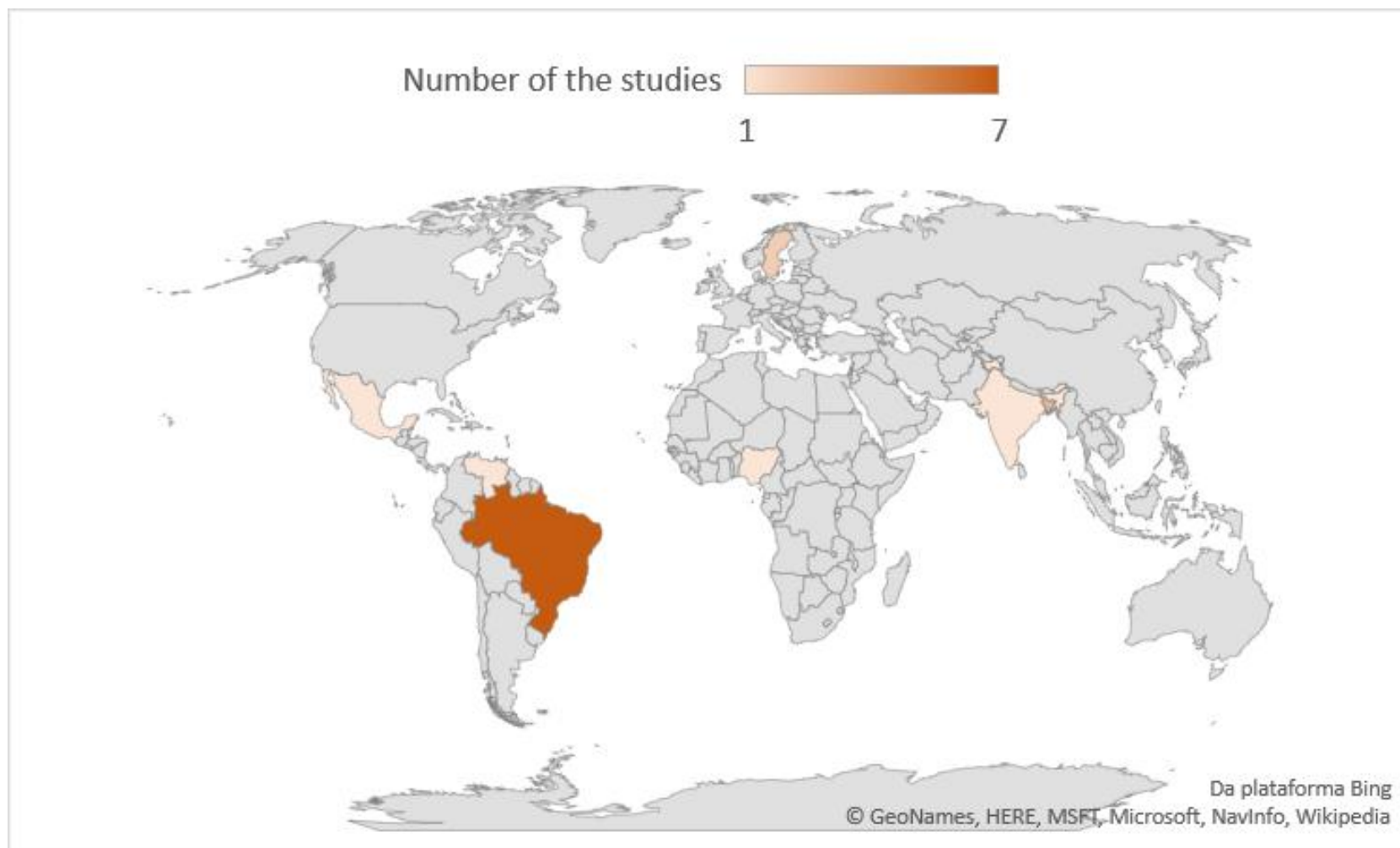

Figure S1. Distribution of studies within countries

Supplement: Supplementary file 1 [file nutrients-11-01222-s001.pdf]
